# Defining the "core microbiome" of the microbial communities in the tonsils of healthy pigs

**DOI:** 10.1186/1471-2180-12-20

**Published:** 2012-02-07

**Authors:** Beth A Lowe, Terence L Marsh, Natasha Isaacs-Cosgrove, Roy N Kirkwood, Matti Kiupel, Martha H Mulks

**Affiliations:** 1 Department of Microbiology and Molecular Genetics and the Center for Microbial Pathogenesis, Michigan State University, East Lansing, MI, USA; 2 Department of Large Animal Clinical Sciences, College of Veterinary Medicine, Michigan State University, East Lansing, MI, USA; 3 Department of Pathobiology and Diagnostic Investigations, College of Veterinary Medicine, Michigan State University, East Lansing, MI, USA; 4 School of Animal and Veterinary Science, University of Adelaide, Adelaide, Australia (Current

## Abstract

**Background:**

Porcine tonsils are the colonization site for many pathogenic as well as commensal microorganisms and are the primary lymphoid tissue encountered by organisms entering through the mouth or nares. The goal of this study was to provide an in-depth characterization of the composition and structure of the tonsillar microbial communities and to define the core microbiome in the tonsils of healthy pigs, using high throughput bar-coded 454-FLX pyrosequencing.

**Results:**

Whole tonsils were collected at necropsy from 12 16-week-old finisher pigs from two healthy herds. Tonsil brushes were also used to collect samples from four of these animals. Bacterial DNA was isolated from each sample, amplified by PCR with universal primers specific for the bacterial 16S rRNA genes, and the PCR products sequenced using pyrosequencing. An average of 13,000 sequences were generated from each sample. Microbial community members were identified by sequence comparison to known bacterial 16S rRNA gene sequences.

The microbiomes of these healthy herds showed very strong similarities in the major components as well as distinct differences in minor components. *Pasteurellaceae *dominated the tonsillar microbiome in all animals, comprising ~60% of the total, although the relative proportions of the genera *Actinobacillus, Haemophilus*, and *Pasteurella *varied between the herds. Also found in all animals were the genera *Alkanindiges, Peptostreptococcus, Veillonella, Streptococcus *and *Fusobacterium*, as well as *Enterobacteriaceae *and *Neisseriaceae. Treponema *and *Chlamydia *were unique to Herd 1, while *Arcanobacterium *was unique to Herd 2.

Tonsil brushes yielded similar results to tissue specimens, although *Enterobacteriaceae *and obligate anaerobes were more frequently found in tissue than in brush samples, and *Chlamydia*, an obligately intracellular organism, was not found in brush specimens.

**Conclusions:**

We have extended and supported our previous studies with 16S clone libraries, using 16S rRNA gene pyrosequencing to describe the microbial communities in tonsils of healthy pigs. We have defined a core microbiome, dominated by *Pasteurellaceae*, in tonsil specimens, and have also demonstrated the presence of unique minor components of the tonsillar microbiome present in each herd. We have validated the use of non-invasive tonsil brushes, in comparison to tonsil tissue, which will facilitate future studies.

## Background

The palatine tonsils of pigs are large, flat, follicular structures on the ventral side of the soft palate, at the junction of the oropharynx and nasopharynx, that are constantly exposed to both ingested and inhaled microorganisms [1]. Both the surface of the tonsils and the extensive tubular tonsillar crypts are an important colonization site for many pathogenic and commensal microorganisms, including both bacteria and viruses [1]. Conversely, the tonsils are also the main oropharyngeal lymphoid tissue, and play a key role in surveillance, detection, and initiation of an immune response against organisms that enter through either the mouth or the nares [1,2].

Asymptomatic carriage in the tonsils provides a reservoir for many bacterial porcine pathogens, such as *Actinobacillus pleuropneumoniae, A. suis, Streptococcus suis, Haemophilus parasuis*, and *Mycoplasma hyopneumoniae*, as well as viral pathogens such as PRRS virus and classical swine fever virus [1,3-8]. Indeed, the tonsils are a routine sampling site in surveillance of many porcine pathogens [1]. Porcine tonsils are also a reservoir for pathogens capable of causing foodborne infections of humans, including *Salmonella *and *Campylobacter *species, *Escherichia coli, Listeria monocytogenes*, and *Yersinia enterocolitica *[9-13]. The commensal tonsillar microbiota likely interacts with these pathogens to either inhibit or enhance colonization and carriage.

In a previous study, standard aerobic culture and culture-independent construction and analysis of 16 s rRNA gene clone libraries were used to examine the microbial communities in the tonsils of healthy pigs [14]. Gram-positive cocci (*Streptococcus, Staphylococcus*, and *Enterococcus *species) predominated in the culture analysis, but were not detected in the clone libraries. In contrast, *Pasteurellaceae *(*Actinobacillus*, *Haemophilus*, and *Pasteurella *species) and anaerobic *Fusobacterium, Bacteroides, Porphyromonas*, and *Prevotella *species were the dominant genera found in the 16S rRNA clone libraries. The goal of the current study was to utilize high throughput bar-coded 454-FLX pyrosequencing to provide a more in-depth characterization of the composition and structure of the tonsillar microbial communities and to define the core microbiome in the tonsils of healthy pigs.

## Methods

### Animals

The study and all animal procedures were approved by the Michigan State University Institutional Animal Care and Use Committee. Eight 18-20 week old pigs from a high health status herd with no recent history of respiratory disease (Herd 1) and four similar pigs from a currently healthy herd with a history of chronic but undefined respiratory problems (Herd 2) were randomly selected for use in this study. Both herds are farrow-to-finish operations weaning at 21 to 24 days of age, with similar management, located in mid-Michigan. Groups of similarly aged pigs were moved from the nursery to the grow-finish rooms all in-all out, although there was a common airspace via either connecting corridor (Herd 1) or connecting doors (Herd 2). Herd 1 Time 1 contains four Hampshire-Yorkshire crossbred pigs (pigs A-D) that were sampled in June 2007. These four pigs received no vaccinations or in-feed antibiotics. Herd 1 Time 2 contains four purebred Yorkshire pigs (pigs J-M) that were sampled 2 years later (April 2009). These pigs received Tylan (Elanco Animal Health, Indianapolis, IN) in-feed and were vaccinated against PCV2. There were no other significant differences in feed, vaccination, or medication between the two sampling periods for Herd 1. Herd 2 contains four Hampshire-Cambrough crossbred pigs (pigs E-H) that were sampled only once, in July 2007. This herd was also vaccinated against PCV2 and received Tylan in-feed. Additionally, Herd 2 received Pulmotil (Elanco Animal Health) until 8 weeks of age. The standard feed ration for Herd 2 was similar to that for Herd 1.

All pigs were taken off feed at least 3 h prior to collection of specimens. Pigs were anesthetized by intramuscular injection of Telazole (6.6 mg/kg) and Xylazine (3.3 mg/kg) prior to transport from the farms to the necropsy facilities at the Michigan State University Diagnostic Center for Population and Animal Health. Pigs were euthanized within 30 min by overdose with a pentobarbital solution (Fatal-Plus, 100 mg/kg, Vortech Pharmaceutical, Dearborn, MI) delivered intravenously into the vena cava, following standard procedures.

Lung specimens from Herd 1 Time 1 pigs A-D and Herd 2 pigs E-H were aseptically sampled and cultured on blood agar and brain heart infusion agar containing 10 μg/ml NAD. No bacterial isolates were recovered from Herd 1 pigs. *Arcanobacterium pyogenes *was recovered from well-defined lesions in the lungs of 1 pig from Herd 2 (pig F).

### Collection of tonsil tissue

The entire right and left palatine tonsils from all 12 pigs were collected. Tonsils were placed into sterile glass Petri dishes where extraneous connective tissue was removed and tonsils were divided into four quarters using a sterile scalpel. One quarter of the right tonsil was combined with one quarter of the left tonsil and this combined sample was minced, placed into a sterile 15 ml conical tube, and immediately frozen at -20°C for subsequent extraction of community DNA.

### Tonsil brush design and use

For this study, we designed a brush for swabbing porcine palatine tonsils. The tonsil brush consisted of three parts: a 0.5″ diameter soft-bristle test tube brush with approximately 5″ of metal handle (VWR International, West Chester, PA), an 8″ long by 0.5″ diameter wooden dowel used as a handle, and a guard for the brush made from 2 15 ml screw capped centrifuge tubes (Fisher Scientific, Pittsburgh, PA). The brush portion was prepared by cutting the brush head to a length of 1″ and sealing the cut end with a drop of superglue to protect the pig and tissue from injury. Once dry, the brush was soaked for 1 h in 10% hydrochloric acid to destroy any contaminating DNA, rinsed thoroughly with sterile H_2_O, and allowed to dry completely. The guard was made by cutting the conical ends off two 15 ml centrifuge tubes, taping the cut ends of the tubes together, and removing one of the caps. The brush was then assembled by inserting the handle of the test tube brush into the end of the dowel and placing the guard over the brush. The guard was secured to the handle with a piece of autoclave tape. Assembled brushes were autoclaved in instant sealing sterilization pouches (Fisher Scientific).

For swabbing porcine tonsils, the cap was removed from the guard and the brush (with the guard still in place) was inserted into the pig's mouth near the tonsil. The guard was then pulled back to expose the brush. Both the right and left tonsils were brushed for approximately 10 s each, with sufficient pressure to allow penetration of tonsillar crypts with the soft brush bristles, and at the same time with care not to cause tissue damage and bleeding. The guard was replaced and brush removed from the pig's mouth. The guard was discarded and the brush removed from the handle and placed into a 50 ml sterile test tube containing 20 ml of ice-cold 80% ethanol. Brushes were then stored at -20°C for subsequent extraction of community DNA.

Tonsil brush specimens were collected from Herd 1 Time 2 pigs (pigs J-M), immediately after euthanasia and prior to removal of the tonsils for tissue collection.

### Isolation of community DNA

Community DNA from pig tonsils was extracted from tonsil tissues using a PowerSoil DNA Isolation Kit (MoBio Laboratories, Carlsbad, CA) as previously described [14]. Community DNA from tonsil brush specimens was extracted using the same kit as follows. The tube containing the tonsil brush and 20 ml of ice-cold 80% ethanol was vortexed vigorously to remove cells and cell debris from the brush. The brush was removed and discarded. The sample in 80% ethanol was divided evenly into 2 sterile Corex^® ^tubes and centrifuged in a refrigerated Sorvall SS-34 rotor at 16,000 × *g *for 30 min. Following centrifugation, supernatants were discarded. One pellet was suspended in 5 ml of ice-cold 80% ethanol and archived at -20°C. The second pellet was suspended in 1 ml phosphate buffered saline (PBS) for DNA extraction. Approximately 0.25 ml of the sample was added to each of 4 MoBio PowerBead tubes. The samples were shaken vigorously in a Bead Beater (BioSpec Products, Bartlesville, OK) for 1.5-2 min at 4°C, and then extracted according to manufacturer's instructions. After purification, the concentration of community DNA was determined spectrophotometrically using a Nanodrop (Thermo Scientific, Wilmington, DE). Fifty percent of the yield was immediately archived at -80°C; the remaining DNA was used for polymerase chain reaction (PCR) amplification and 454 pyrosequencing.

### 454 pyrosequencing

For 454 Flx sequencing, community template DNAs were amplified with primers designed by the Ribosomal Database Project (RDP) at Michigan State University [15]. The forward primer contains the Flx-specific terminal sequence (5'-GCCTCCCTCGCGCCATCAG-3') followed by a six base tag and then the 16S rRNA-specific 3' terminus of the composite primer (5'-AYTGGGYDTAAAGNG-3'). The reverse primer was composed of four variants targeting the same 16S rRNA region to maximize coverage of the database (R1 = /5'/TACNVGGGTATCTAATCC; R2 = /5'/TACCRGGGTHTCTAATCC; R3 = /5'/TACCAGAGTATCTAATTC; R4 = /5'/CTACDSRGGTMTCTAATC). The 3' terminus of the forward primer is at *E. coli *position 578 and the 3' terminus of the reverse primer is at position 785. Pilot scale (25 μl) PCR reactions for optimization were followed by 2-3 preparative 50 μl amplification reactions. High fidelity Taq (Invitrogen Platinum) was used with MgSO4 (2.5 mM), the vendor supplied buffer, BSA (0.1 mg/ml), dNTPs (250 μM) and primers (1 μM). A three minute soak at 95°C was followed by 30 cycles of 95°C (45 s), 57°C (45 s) and 72°C (1 min) with a final 4 min extension at 72°C. PCR products were agarose gel purified (2% metaphor in TAE) and bands were extracted with a QIAquick Gel Extraction Kit (Qiagen, Valencia, CA). Gel extracted material was further purified with a Qiagen PCR Cleanup kit. Quantification of purified PCR product was with PicoGreen using Qubit (Invitrogen, Carlsbad, CA). The PCR products from 20 to 40 samples were combined in equal mass amounts and loaded into a Roche GS Flx system using vendor specified chemistries.

### Sequence analysis tools

All sequences derived from 454 Flx sequencing were processed through the RDP pyrosequencing pipeline [15-17]. Initial processing included screening and removing short reads (those lacking both primer sequences) and low quality reads (any with errors in the primer sequence). Sequences were sorted based on sequence tags and trimmed of primer and tag sequences. The remaining high quality sequences were taxonomically identified using the Classifier tool at a 60% confidence level. The classifier output was then used for analysis of similarities and difference between herds (Additional files 1, 2, 3, 4, 5). For analysis of the data at the genus level, all genera with fewer than 5 representatives were dropped from the analysis.

To identify members of the family *Pasteurellaceae *and genus *Streptococcus *to the lowest possible phylogenetic level, we obtained all the 138 near full-length type sequences from family *Pasteurellaceae *and genus *Streptococcus *from RDP release 10.22 (August 2010). We also added sequence AF486274 ("*Actinobacillus porcitonsillarum*"). These 139 sequences were aligned by the Infernal aligner [16] trained by RDP [17]. The final reference set contained the region corresponding to the 454 FLX amplicon (*E. coli *position 578 to 784) sliced from the alignment. To determine the nearest neighbor, the 454 FLX sequences passing the RDP Pyro initial filtering were aligned by the Infernal aligner and the distance between each FLX sequence and reference sequences was calculated. The reference sequence with the closest distance was reported. In case of tie, all the reference sequences were reported.

### Statistical analysis

For the statistical analyses of sequences, we used a 0.03% cutoff value for clustering. This is consistent with previous analyses of 454 data [18] as well as the historical value frequently used over the past 15 years [19,20]. Similarly we used this cutoff in evaluating members of family *Pasteurellaceae *and genus *Streptococcus*. For comparative statistical analyses, aligned sequences were clustered using the RDP Complete Linkage Clustering Tool and the resulting cluster files were used to calculate Jaccard and Sørensen indices [17]. For comparative statistical analyses, aligned sequences were clustered using the RDP Complete Linkage Clustering Tool and the resulting cluster files were used to calculate Jaccard and Sørensen indices [17]. Cluster files were also reformatted with the EstimateS Formatter Tool through the RDP website. Principle component analysis followed by centroid calculations with a 95% confidence limit were performed in R (version 2.10; http://www.r-project.org/) with Vegan package (http://vegan.r-forge.r-project.org) using the EstimateS formatted files. Chao 1 was calculated using the cluster files derived from each sample and from merged samples for herds using the RDP Pyrosequencing Pipeline. Simpson's Diversity index was calculated with MOTHUR [21].

## Results

Community DNA was isolated from whole tonsil tissue (Pigs A-M) or tonsil brushings (Pigs J-M) as described in Methods. Tonsil tissue samples were collected in spring 2007 from two different herds, and again in spring 2009 from Herd 1. Tonsil brush samples were collected only in spring 2009 from Herd 1, to test the validity of this rapid and painless sampling technique in comparison to euthanasia and removal of tonsil tissue. Sixteen samples (four groups of four samples) were collected and analyzed by 454 Flx pyrosequencing, and comparisons were made between Herd 1 and Herd 2, between Herd 1 Time 1 and Herd 1 Time 2 tissues, and between tissue and brush samples from Herd 1 Time 2 in these results.

### Bar-coded 16S pyrosequencing

A total of 210,433 quality reads were obtained from the four groups of pigs sampled, with at least 15,000 reads per group. Samples of tonsil tissue from Herd 1 at time 2 yielded the fewest number of quality reads. Table 1 shows the number of reads obtained from each of the four groups of pigs and the percent of those reads that could be taxonomically assigned at a 60% confidence level using the RDP Classifier. Overall, greater than 97% of the total reads could be taxonomically assigned at the phylum, class, and order level. This dropped to 90.5% at the family level and further dropped to 72.3% at the genus level. Taxonomic assignment of reads was consistently lower at all levels for Herd 2 compared to all three groups of samples from Herd 1.

**Table 1:** Taxonomic characterization of tonsillar microbial communities

	Sample	# Reads^a^	Phylum^b^	Class^b^	Order^b^	Family^b^	Genus^b^
**Herd 2**	**Tissue**	99894	95.6%	95.4%	94.8%	82.7%	64.7%

**Herd 1 Time 1**	**Tissue**	54932	99.7%	99.6%	99.1%	96.7%	85.0%

**Herd 1 Time 2**	**Tissue**	15929	99.8%	99.5%	99.4%	98.7%	70.1%

**Herd 1 Time 2**	**Brush**	39678	99.9%	99.5%	99.5%	98.6%	75.0%

**Total # reads**		210433	205795	205346	204467	190540	152192

**Avg % Assigned**			**97.8%**	**97.6%**	**97.2%**	**90.5%**	**72.3%**

^a ^the sum of all sequences of 4 individuals

^b^% of reads taxonomically assigned at each level

**Figure 1:**
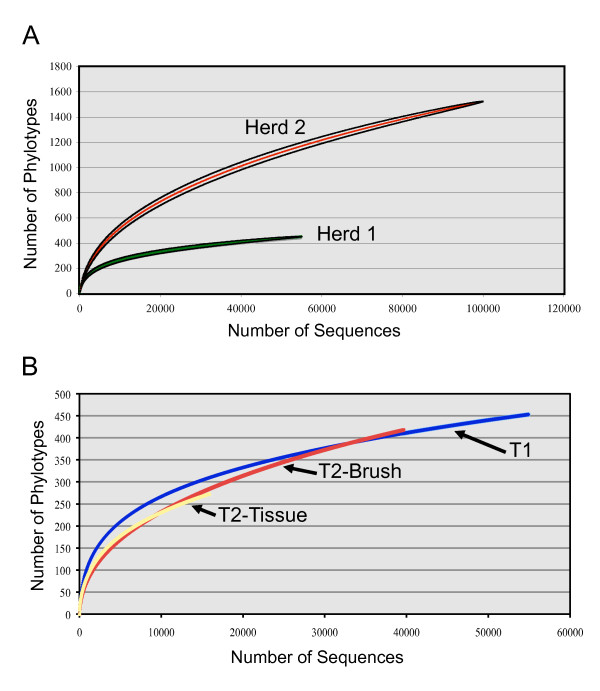
**Rarefaction curves computed with the RDP Pyrosequencing Pipeline**. Rarefaction curves are presented for each group of samples obtained by 454 pyrosequencing. The curves for herds 1 and 2 at time 1 are shown in panel A, while the curves for all three groups of samples from herd 1 are shown in panel B.

Figure 1 shows the rarefaction plots for the four groups. Herd 1 and Herd 2 plots demonstrate that Herd 2 had significantly more phylotypes and greater unsampled diversity (Figure 1A). Comparison of the three groups of Herd 1 pigs reveals similar trajectories even though the number of reads sampled varied (Figure 1B). Taken together, this suggests that the microbial community in the tonsils in Herd 2 was more complex at this level of interrogation.

As stated above, a total of 210,433 reads was obtained for the four groups. Table 2 indicates the number of reads made from each individual sample as well as the total for each group. The number of reads for each individual and each group forms the basis of the comparisons for the number of OTUs, Chao-1 richness, and the Simpson diversity indices. Using a cutoff of 97% identity for species level distinctions, the number of OTUs detected per sample ranged from 57 to 730. In general, the number of OTUs detected increased with the number of reads, though there was no direct proportionality. Herd 2 showed the greatest variability across the four pigs sampled ranging from 197 to 730 OTUs. Pigs from Herd 1, regardless of sampling time or method, had less diversity and variation across pigs (57-251 OTUs). The first sampling that compared Herd 1 with Herd 2 had roughly twice as many sequences in Herd 2 as in Herd 1 (99,894 vs 54,932). However, the number of detected OTUs and Chao 1-estimated OTUs of Herd 2 were 3.3 and 4 fold greater than those of Herd 1. The Shannon and Simpson's Diversity Indices reflected the trends seen with detected and estimated richness, with Herd 2 measurably more diverse than Herd 1. This could be seen in the rank abundance curves (data not shown) where Herd 2 had greater asymmetry (less even) and a longer tail comprised of OTUs with small populations. The Simpson's evenness measurement indicated that all communities were quite uneven (1.0 = perfect evenness) but that the second sampling of Herd 1 derived from extracted tissue was less skewed than other communities.

**Table 2:** Diversity and richness of the tonsillar microbial communities

	# Reads	# OTUs^a^	Chao-1^b^	Shannon^c^	Simpson^d^	Simpson evenness^e^
**Pig E**	43770	582	980	3.14	0.10	0.02

**Pig F**	11386	197	268	3.40	0.07	0.07

**Pig G**	16519	485	820	3.73	0.05	0.04

**Pig H**	28219	730	1224	3.42	0.11	0.01

**Herd 2 Time 1**	**99894**	**1525**	**2513**	**3.58**	**0.06**	**0.01**

**Pig A**	12268	128	161	2.37	0.21	0.03

**Pig B**	14885	190	235	3.17	0.09	0.05

**Pig C**	9392	182	237	2.81	0.14	0.04

**Pig D**	18387	135	291	3.23	0.07	0.11

**Herd 1 Time 1**	**54932**	**453**	**628**	**3.23**	**0.07**	**0.03**

**Pig J**	5523	122	191	3.26	0.07	0.12

**Pig K**	2760	67	88	2.70	0.11	0.14

**Pig L**	6295	167	233	3.12	0.09	0.06

**Pig M**	1351	57	87	2.45	0.15	0.11

**Herd 1 Time 2**	**15929**	**273**	**382**	**3.23**	**0.08**	**0.05**

**Pig J Brush**	13361	155	228	2.04	0.29	0.02

**Pig K Brush**	5672	102	141	2.38	0.14	0.07

**Pig L Brush**	9380	251	465	2.35	0.26	0.01

**Pig M Brush**	11265	136	164	2.83	0.11	0.06

**Herd 1 Brush**	**39678**	**418**	**650**	**2.53**	**0.18**	**0.01**

^a ^number of OTUs (based on 0.03 cut-off) found in each sample or herd

^b ^the estimated richness of an environment based on 0.03 cut-off

^c ^computed at the RDP Pyrosequencing Pipeline

^d ^calculated with MOTHUR [21] using a distance matrix computed at RDP Pyrosequencing Pipeline

^e ^derived from Simpson's Index where E = (1/D)/S, D is the Simpson's Index and S is the total number of species (OTUs)

### Phylum, class, and order level structure of the tonsillar communities

We found members of 17 different phyla of bacteria in one or more tonsil specimens examined (Additional file 1). Microbial communities in all pigs in all four groups of samples were dominated by *Proteobacteria*, which averaged 73.4% of the communities (ranging from 47.0% to 94.5% in individual specimens); *Firmicutes*, which averaged 17.8% (ranging from 3.1% to 45.6%); and *Fusobacteria*, which averaged 5.6% (ranging from 0.6% to 16.3%) of the total reads assigned. Together, the *Proteobacteria, Firmicutes*, and *Fusobacteria *comprised 96.8% (ranging from 87.5% to 99.6% in individual specimens) of the microbial communities in these samples. *Actinobacteria *(1.2%) and *Bacteroidetes *(0.8%) were also found in most pigs in all four groups of samples. These five phyla form the core microbiome of porcine tonsils, and together comprised on average 98.8% (ranging from 89.5% to 100%) of the reads assigned to the phylum level (Table 3). In addition, *Tenericutes *(0.03%) were found in small numbers in at least one pig in each group of samples.

**Table 3:** The core microbiome of porcine tonsils

Phylum	% of total	Class	% of total	Order	% of total	Family	% of total	Genus	% of total
*Proteobacteria*	73.4	*Gammaproteobacteria*	69.8	*Pasteurellales*	56.0	*Pasteurellaceae*	60.2	*Actinobacillus*	37.0

								*Haemophilus*	6.6

								*Pasteurella*	16.1

				*Pseudomonadales*	11.8	*Moraxellaceae*	12.3	*Alkanindiges*	12.0

				*Enterobacteriales*	2.0	*Enterobacteriaceae*	2.2		

		*Betaproteobacteria*	3.2	*Burkholderiales*	0.3				

				*Neisseriales*	2.8	*Neisseriaceae*	3.0		

		*Alphaproteobacteria*	0.3						

*Firmicutes*	17.8	*Clostridia*	14.3	*Clostridiales*	14.3	*Peptostreptococcaceae*	2.2	*Peptostreptococcus*	2.6

						*Veillonellaceae*	4.4	*Veillonella*	3.2

		*Bacilli*	3.5	*Lactobacillales*	3.4	*Streptococcaceae*	0.5	*Streptococcus*	0.6

*Fusobacteria*	5.6	*Fusobacteria*	5.6	*Fusobacteriales*	5.6	*Fusobacteriaceae*	5.6	*Fusobacterium*	7.0

*Actinobacteria*	1.2	*Actinobacteria*	1.2	*Actinomycetales*	0.9				

*Bacteroidetes*	0.8	*Bacteroidia*	0.3	*Bacteroidales*	0.3				

5/17 phyla identified	98.8	8/27 classes identified	98.2	10/34 orders identified	97.4	8/61 families identified	90.4	8/101 genera identified	85.1

NOTE: Almost half of the *Clostridiales *could not be assigned at the family level, and > 92% of the *Neisseriaceae *could not be assigned to a genus.

Distribution at the class level followed well from the phylum level data. We found members of 27 different classes of bacteria in at least one of the tonsil specimens (Additional file 2). Classes found in all animals in all four groups of specimens included, in order of prevalence, G*ammaproteobacteria *(69.8% of the total reads taxonomically assigned at the class level), *Clostridia *(14.3%), *Fusobacteria *(5.6%), *Bacilli *(3.5%), and *Betaproteobacteria *(3.2%). *Actinobacteria *(1.2%), *Alphaproteobacteria *(0.3%), and *Bacteroidia *(0.3%) were found in most animals in all groups of samples. These eight classes form the core microbiome of porcine tonsils, and together represent 98.2% (ranging from 89.2% to 99.9% in individual specimens) of the total reads assigned at the class level (Table 3). In addition, *Epsilonproteobacteria *(0.1%), and *Mollicutes *(0.02%) were found at least one animal in each group. Both *Deltaproteobacteria *(0.1%) and *Sphingobacteria *(0.1%) were found in at least one animal in all three groups of tissue specimens but not in the brush specimens.

We found members of 34 different orders of bacteria in at least one tonsil specimen (Additional file 3). The predominant orders found in all four groups of specimens included *Pasteurellales *(56.0%), *Clostridiales *(14.3%), *Pseudomonadales *(11.8%), *Fusobacteriales *(5.6%), *Lactobacillales *(3.4%), *Neisseriales *(2.8%) and *Enterobacteriales *(2.0%). In addition, the *Actinomycetales *(0.9%), *Burkholderiales *(0.3%), and *Bacteroidales *(0.3%) were found in most animals in all groups of specimens. These ten orders form the core microbiome of porcine tonsils, and together represent 97.4% (ranging from 88.0% to 99.7% in individual specimens) of the reads assigned at the order level (Table 3). *Bacillales *(0.14%) and *Campylobacterales *(0.13%) were also found in small numbers in half of the specimens.

### Family and genus level structure of the tonsillar communities

We found members of 61 families (Additional file 4) and 101 genera (Additional file 5) in at least one tonsil specimen. Five families were found in all pigs in all groups of specimens: *Pasteurellaceae *(60.2%), *Moraxellaceae *(12.3%), *Fusobacteriaceae *(5.6%), *Veillonellaceae *(4.4%), and *Neisseriaceae *(3%). In addition, three families, the *Peptostreptococcaceae *(2.2%), *Enterobacteriaceae *(2.2%), and *Streptococcaceae *(0.5%), were found in most animals in all groups of specimens. These eight families form the core microbiome in porcine tonsils, and represent 90.4% (ranging from 73.5% to 99.0% in individual specimens) of the reads assigned at the family level (Table 3). It should be noted that almost half (46.8%) of the *Clostridiales *could not be assigned at the family level.

**Figure 2:**
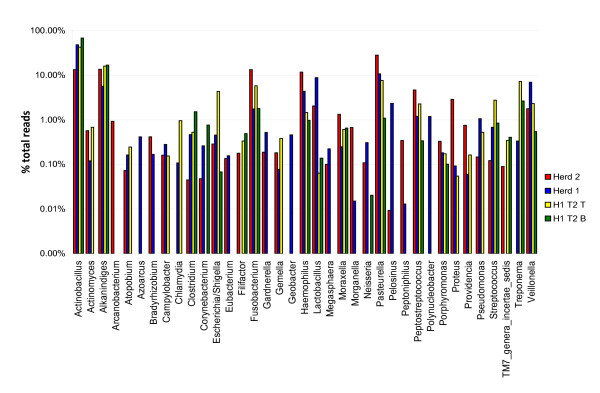
**Taxonomic characterization of the four groups of samples obtained by 454 pyrosequencing**. Bars illustrate the proportion of reads classified into particular genera. Only genera that contain at least 0.01% of the total number of reads are shown.

Of the 101 genera identified in these samples, 49 were found in both herds (Additional file 5). Thirty-seven genera represented at least 0.1% of the total reads from all specimens (Figure 2). Of these 37 genera, 13 were found in all 4 groups of specimens, 2 were found only in Herd 1, 1 was found only in Herd 2, and 8 were found in tissue specimens but not in brush specimens.

**Figure 3:**
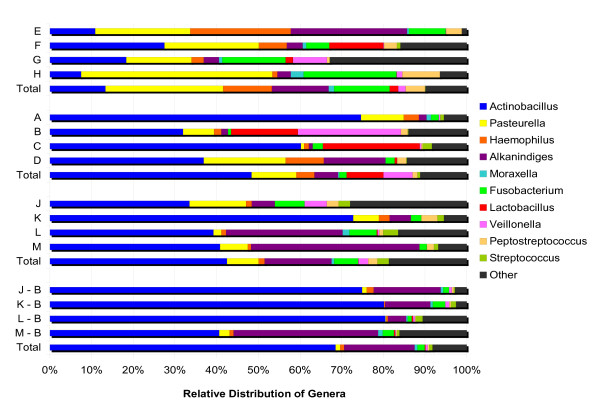
**Relative distribution of the ten most abundant genera identified**. The distribution of genera in each individual pig, as well as the group totals are shown.

The relative distribution of the top ten genera found in these specimens is shown in Figure 3. These 10 genera comprised on average 88.3% (ranging from 67.2% to 98.8%) of the total genera in the microbial communities in these specimens. *Actinobacillus (Pasteurellaceae), Alkanindiges (Moraxellaceae*), *Fusobacterium (Fusobacteriaceae*), and *Haemophilus (Pasteurellaceae*) were found in all pigs in all groups of specimens. *Pasteurella (Pasteurellaceae*), *Veillonella (Veillonellaceae)*, *Peptostreptococcus (Peptostreptococcaceae)*, and *Streptococcus (Streptococcaceae) *were found in almost all pigs in all groups of specimens. These eight genera form the core microbiome in porcine tonsils, and represent 85.1% of the reads assigned to the genus level (Table 3). In addition, *Moraxella (Moraxellaceae) *and *Lactobacillus (Lactobacillaceae) *were found in more than half of the samples tested, and are included in the top ten genera shown in Figure 3. It should be noted that most (> 92%) of the *Neisseriaceae *could not be assigned at the genus level.

### Species level structure of tonsillar communities

We utilized a pairwise distances program to compare the 454 16S sequences from each pig to the V4 (variable region 4) regions of the type strains for species in the families *Pasteurellaceae *and *Streptococcaceae*. Using a 97% cutoff, we determined the closest affiliation for each sequence.

Sequences with closest affiliations to *Actinobacillus indolicus, A. minor, "A. porcitonsillarum"*, and *Haemophilus parasuis *were found in all samples. Sequences with closest affiliation to *A. porcinus, A. rossii, H. felis, Pasteurella aerogenes, P. canis, P. multocida*, and *Streptococcus suis *were found in most samples. Finally, sequences with closest affiliation to *S. plurextorum, A. lignieresii*, and *A. seminis *were found in small numbers in 40% of the samples.

### Comparison of Herd 1 time 1 and time 2 communities

To determine whether the microbial communities in a given swine herd change over time, we compared the communities in tonsil tissue from pigs from Herd 1 sampled two years apart, in 2007 (time 1) and 2009 (time 2). Overall, the core microbiome of the two groups of samples remained quite similar at the phylum, class, order, and family levels, with the exception that *Neisseriales *were more frequently identified at time 2 (10.1% of the total) than time 1 (0.6%) (Additional file 3) and *Lactobacillaceae *were more common at time 1 (7.8% of the total) than time 2 (0.04%) (Additional file 4). Both were dominated by *Pasteurellaceae*, which comprised 64.2% of the total at time 1 and 50.3% at time 2 (Additional file 4). The distribution of the top ten genera was very similar, with the exception that *Lactobacillus *was much more common at time 1 than time 2 (Figure 3). Both groups of samples also contained the genera *Treponema *(phylum *Spirochaetes*) and *Chlamydia *(phylum *Chlamydiae*), with higher numbers of both seen at time 2. In addition, all Herd 1 time 1 samples also contained the genus *Pelosinus *(family *Veillonellaceae*), which averaged 2.3% of the total in Herd 1 time 1 but was not found at time 2 (Additional file 5). No genus present in most animals in the sample were identified as unique to Herd 1 time 2. There were no significant differences between the clusters at a 97% cutoff aligned to species of *Pasteurellaceae *and *Streptococcaceae *identified in the two groups of Herd 1 samples.

There were a variety of organisms associated with soil and water, such as *Polynucleobacter, Geobacter*, and *Azoarcus*, that were found only in Herd 1 at time 1, and generally only in one or two animals (Additional file 5). Indeed, Herd 1 time 1 samples contained members of 6 phyla, *Chloroflexi, Cyanobacteria, Gemmatimonadetes, Planctomycetes, Synergistetes*, and *Verrucomicrobia*, which together comprised 0.5% of the total tonsillar communities in Herd 1 time 1 samples but were not found in time 2 samples from herd 1 (Additional file 1).

### Comparison of Herd 1 time 1 and Herd 2 communities

The microbial communities of Herd 1 time 1 and Herd 2 tissue samples showed strong similarities in the core microbiomes as well as distinct differences. In both herds, the tonsillar microbiomes were dominated by *Pasteurellaceae *(64.2% of Herd 1, 57.4% of Herd 2). However, the distribution of genera within that family varied between the herds; 75% of the *Pasteurellaceae *in Herd 1 were identified as genus *Actinobacillus*, while in Herd 2, 50% of the *Pasteurellaceae *were identified as genus *Pasteurella *(Figure 3 and Additional file 5). Reads identified as genus *Fusobacterium *formed a larger percentage of the total in Herd 2 (13.3%) than Herd 1 (1.7%). Distribution of the remaining major genera in the core microbiome was similar in the two herds (Figure 3).

Of the 101 genera identified, 41 were unique to Herd 1 and 11 were unique to Herd 2 (Additional file 5). Of those genera unique to Herd 1, only 2, *Treponema *(phylum *Spirochaetes) *and *Chlamydia *(phylum *Chlamydiae*) were found in most pigs from Herd 1. Reads identified as *Treponema *were found in all three groups of Herd 1 samples, although in smaller numbers at time 1 (0.3% of the total) than at time 2 (an average of 3.9% from tissue and brush samples), but were not found in Herd 2 (Figure 2). Reads identified as *Chlamydia *comprised on average 0.3% of the total reads in both groups of Herd 1 tissue specimens but were not found in brush specimens (Figure 2).

Of the 11 genera unique to Herd 2, only *Arcanobacterium *(phylum *Actinobacteria*, family *Actinomycetaceae) *was found in all animals. Reads identified as *Arcanobacterium *comprised 0.9% of Herd 2, but were not found in any Herd 1 specimen (Figure 2). This was the only genus unique to Herd 2 that was found in most animals and represented ≥ 0.1% of the total genera identified in all specimens. In addition, reads assigned to proposed phylum SR1 comprised 0.05% of Herd 2 but were not found in Herd 1.

At the 97% cutoff, both Herd 1 and Herd 2 contained the same core clusters of *Pasteurellaceae *and *Streptococcaceae*, although the relative proportions varied between the two groups of samples. For example, Herd 1 contained a higher fraction of sequences most closely affiliated with *A. minor *and fewer affiliated with *A. porcitonsillarum *than Herd 2. Furthermore, sequences most closely related to *Streptococcus plurextorum *and *S. thermophilus *were found in most samples from Herd 1, but not Herd 2.

### Comparison of communities in Herd 1 time 2 tissue and brush samples

The predominant families (with ≥ 1% of the total assigned reads) and genera in both tissue and brush specimens from Herd 1 time 2 are the *Pasteurellaceae *(*Actinobacillus, Pasteurella, Haemophilus*); *Moraxellaceae (Alkanindiges, Moraxella); Neisseriaceae *(not assigned at the genus level)*; Spirochaetaceae (Treponema); Veillonellaceae *(*Veillonella*)*; Fusobacteriaceae *(*Fusobacterium*); *Streptococcaceae (Streptococcus) *and *Peptostreptococcaceae (Peptostreptococcus*, *Filifactor) *(Figure 2).

*Enterobacteriaceae *(several different species) and obligate anaerobes were more frequently found in tissue than in brush samples (Figures 2 and 3; Additional files 4 and 5). *Chlamydia*, an obligately intracellular organism, comprised 0.95% of the reads assigned to the genus level in the tissue specimens, but was not found in the brush specimens (Additional file 5). Other differences generally reflect either a very small number of reads or reads from only 1-2 samples (Additional files).

### Statistical comparison of communities

**Figure 4:**
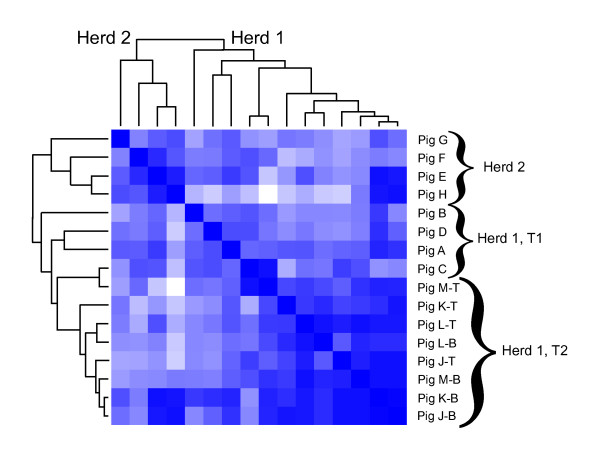
**Jaccard indices of pig tonsil communities**. Indices are presented clustered and plotted in heat map format where light to dark indicates increasing similarity.

Figure 4 shows the Jaccard analysis of the clustered sequences from each tonsil community. The samples from Herd 1 and Herd 2 from the same year (Time 1, 2007) are clearly distinguishable. Samples from Herd 1 taken 2 years later (Time 2, 2009) group with samples taken in time 1 from Herd 1, but are distant from Herd 2. The Jaccard indices of the time 2 sampling from Herd 1 where community samples were derived from both tonsil tissue and brushed tonsils indicate high similarity between these two sampling methods. Some variability exists within the Herd 1 Time 2 samples, as indicated by Pigs K and J from the brush samples where substantial similarities exist with at least two pigs from Herd 2 (lower left of Panel A).

**Figure 5:**
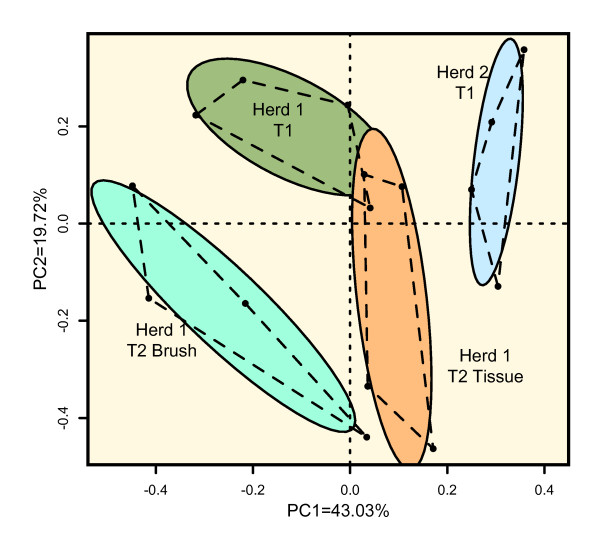
**Principle Component Analysis (PCA) results on all individuals sampled**. PCA was performed at the level of OTUs, clustering sequences at a 3% difference. The PCA plot of tonsillar communities shows PCA analysis using the first two components, accounting for 62.75% of the sample variation. Each point represents the tonsillar community of one individual pig. Colored circles represent the 95% confidence limit for each group of samples.

Principle component analysis (PCA), using the first two factors (PC1 and PC2) was performed using communities from each pig sampled (Figure 5). Each point represents one tonsil community while the colored areas represent the 95% confidence limit of each group. Using the first two components explains 63% of the total variation among the individual samples. This demonstrated that the microbial communities were distinguishable from one another, but relatively close in phylogenetic space as judged by the range of eigenvalues.

## Discussion

We have previously reported the first culture-independent analysis of the microbial communities of the tonsils of healthy pigs [14]. In the previous study, we analyzed 831 16S rRNA gene sequences from clone libraries constructed from samples from eight pigs from two healthy herds. In the current study, we analyzed the same samples (Herd 1 Time 1 and Herd 2 samples), as well as additional samples collected from Herd 1 two years after the first set by two different collection methods (Herd 1 Time 2 tissue and brush samples), using high through-put next generation sequencing methods to generate 210,433 sequences to extend our initial community analysis to greater depth.

Overall, we found strong similarities between the four groups of samples as well as minor unique differences. We identified a "core microbiome" for porcine tonsils that includes eight core genera from six core families (*Pasteurellaceae, Moraxellaceae, Fusobacteriaceae, Veillonellaceae, Peptostreptococcaceae*, and *Streptococaceae*) as well as members of the *Enterobacteriaceae*, which varied in genera found from sample to sample, and *Neisseriaceae*, which could not be identified to the genus level (Table 3). Two additional genera, *Moraxella *and *Lactobacillus*, that are included in the ten most abundant genera identified (Figure 3) were found less consistently, and in particular were missing from most of the Herd 1 Time 2 tissue specimens, and therefore are not included in the core microbiome that we have defined as "found in most animals in all groups".

As in the previous study [14], *Pasteurellaceae (Actinobacillus, Haemophilus*, and *Pasteurella *species) dominated the tonsillar microbial communities in all pigs examined, comprising on average 60.2% of the total reads, and ranging from 39.2% to 87.0% in individual pigs. The distribution of genera within the family *Pasteurellaceae *- with *Actinobacillus *predominate in Herd 1 samples and *Pasteurella *in Herd 2-also compares well with the previous study.

However, a major difference between the results of the two studies is the glaring lack of *Bacteroidetes *in the current data. In the previous study [14], sequences identified as belonging to the order *Bacteroidales *(genera *Bacteroides, Prevotella*, and *Porphyromonas*) comprised the second most dominant group (30% of the sequenced clones) after the *Pasteurellales*, and were found in almost all animals. Three additional species of *Porphyromonadaceae *(*Dysgonomonas*, *Parabacteroides*, and *Tannerella) *were found in a few animals, particularly from Herd 2. In contrast, *Bacteroidales *comprised 0.3% of the sequence reads in the current study, including among the Herd 1 time 1 and Herd 2 samples that were the identical samples used in the previous study. An unexpectedly low abundance of *Bacteroidetes *has been found in other studies using high-throughput bar-coded pyrosequencing [22-24]. One potential explanation cited is variation in the samples analyzed [22-24], which is not the case in our study. These were the same DNA samples used in the previous study [14]. A second explanation would be partial degradation of these samples, resulting in loss of *Bacteroidetes *DNA. However, these same samples have also recently been analyzed with 454-Titanium primers and shown to still contain *Bacteroidetes *DNA (M. H. Mulks & T. L. Marsh, unpublished observations). A more likely explanation is amplification bias during the PCR reactions. However, all of the primer sets used in these studies, which targeted three different variable regions of the 16S gene-the V4 region in the current study, V5 [22], and V6 regions [23,24]-were shown in silico to cover the *Bacteroidetes *species, and the V4 primers were tested experimentally against genomic DNA from known *Bacteroides *isolates and shown to amplify 16 s rDNA. It is likely that members of the *Bacteroidetes *are also part of the core microbiome of porcine tonsils, despite the lack of evidence in our current data.

While there were clear and strong similarities between the core microbiomes of all of the groups examined, there were also unique differences in minor genera found or missing from particular groups. These differences can not readily be explained by differences in overall herd management or antibiotic usage in the groups (no antibiotics in Herd 1 time 1, Tylan in Herd 1 time 2, and Tylan plus Pulmotil in Herd 2). For example, reads identified as *Arcanobacterium *were found in all Herd 2 samples, and comprised 0.93% of the reads from that herd, but were not found in any Herd 1 sample. In contrast, reads identified as *Treponema *were found in all but one sample from Herd 1, but not in any sample from Herd 2, and *Chlamydia *were found in Herd 1 tissue samples but not in Herd 2 samples. *Lactobacillus *was abundant in most samples from both Herd 1 time 1 and Herd 2, but was rare in Herd 1 time 2 samples. *Pelosinus *was abundant only in Herd 1 time 1, not Herd 2 or Herd 1 time 2 samples. There were many other genera found in small numbers in 1-2 animals per group that were unique to that group, such as *Polynucleobacter *and *Geobacter *in Pig D from Herd 1 time 1 (Additional file 5), but no others that could be found in most animals in one group but not in animals of another group. These results indicate that, despite the small sample number, we can identify differences in the minor genera found in the two different herds.

One goal of this project was to test tonsil brushes as an alternative, non-invasive method to collect tonsil samples, eliminating the need to euthanize animals to collect tonsil tissue. The Jaccard analysis (Figure 4) clearly indicated that all samples from the second sampling of Herd 1 were more similar to each other than to samples from Herd 1 and 2. We could detect differences between the brush and tissue extraction procedures as indicated in Figure 5, but the difference was small based on the range of eigenvalues. The detected statistical differences were a consequence of an increase in the percentage of reads identified as *Actinobacillus*, fewer sequences of *Fusobacterium, Veillonella*, and *Peptostreptococcus*), and no detectable sequences from the obligate intracellular pathogen *Chlamydia *in the brush specimens. These results indicate that use of tonsil brushes provides a slightly different but sufficiently representative picture of the microbial community that is suitable for within or across herd comparisons when compared to direct extraction of tonsil tissue.

A wide range of bacterial and viral porcine pathogens are routinely isolated from the tonsils [1]. In this study, we identified large numbers of sequences whose closest affiliate in the database were *Haemophilus parasuis *and *Pasteurella multocida *(on average 12.8% and 9.3%, respectively, of reads identified at the 97% cutoff), as well as small numbers of sequences closest to *Streptococcus suis *(on average 0.4% of reads identified) from almost all samples. However, we did not find sequences affiliated with *Actinobacillus pleuropneumoniae *or *A. suis*, which have been reported to be found in most swine herds in Ontario, Canada [7]. Small numbers of sequences closest to *Mycoplasma *were found in a few pigs, but these were not identified beyond the Classifier function of the RDP. Herd 1 has been regularly tested and found to be free of *A. pleuropneumoniae, A. suis*, and *Mycoplasma*, which is substantiated by these results. We were surprised not to find sequences consistent with the presence of pathogenic *Actinobacillus *species in Herd 2, which has had a history of chronic but undefined respiratory problems. It is possible that these chronic problems are related to the higher numbers of *Pasteurella sequences *found in Herd 2, or to the presence of another known respiratory pathogen, *Arcanobacterium*, found in Herd 2 but not Herd 1.

In addition to porcine pathogens, many bacterial agents of foodborne infections of humans have been isolated from pig tonsils, including members of the *Enterobacteriaceae *such as *Salmonella *species, *Escherichia coli*, and *Yersinia enterocolitica *as well as *Campylobacter *species and *Listeria monocytogenes *[9-13]. We found low numbers of *Campylobacter *(0.17% of total reads) and *Escherichia *(0.59% of total reads) in most of the pig tonsils in this study. In addition, we found other *Enterobacteriaceae *(1.9% of the total) that are rarely associated with human foodborne illness, including *Citrobacter, Enterobacter, Morganella, Proteus*, and *Providencia*, in one or more pigs. We did not find *Salmonella, Yersinia*, or *Listeria *in these tonsil samples from healthy pigs.

The only other mammalian system where the tonsillar microbiota has been reported is in humans. Culture-based studies of human tonsils have identified *Streptococcus pyogenes; S. pneumoniae; *Group C, F, and G β-hemolytic streptococci; several α-hemolytic and non-hemolytic streptococci; *Staphylococcus aureus; Haemophilus influenzae; H. parainfluenzae; *and *Moraxella catarrhalis *in aerobic cultures [25-31]. Many species of the *Bacteroides-Prevotella-Porphyromonas *group, *Fusobacterium, Lactobacillus, Peptostreptococcus*, and *Veillonella *have also been isolated using anaerobic cultures. Culture-independent analyses using 16S rRNA gene sequences, by clone libraries [31,32], PhyloChip microarray [32], and pyrosequencing [22], have all supported the predominance of members of the phylum *Firmicutes *in human tonsils/oropharynx, with *Streptococcus mitis, S. australis, S. parasanguinis, S. intermedius, Gemella sanguinis, G. haemolysans, Granulicatella adjacens*, and *Gr. elegans *most commonly found [31,33]. *Proteobacteria *and *Bacteroidetes *were also commonly identified by 16S sequencing techniques [31-33]. In strong contrast to our results with porcine tonsils, *Pasteurellaceae *were identified as a very small percent of the human tonsillar or oropharyngeal community by culture-independent methods [31,32].

We recognize that the short reads derived from 454-Flx limit the phylogenetic information. However previous workers have reported that short reads suffice (at some level) for community analyses [18,34]. As next generation sequencing improves and the read lengths grow, short reads will become less of an issue.

We also recognize that this study is limited to four animals from one herd and eight from a second herd, all healthy animals from two farms that are geographically close and have similar management styles. Clearly this is a preliminary "core microbiome" that will likely evolve as samples from more diverse herds are analyzed. With this limitation, the strong similarities seen between samples suggest that next generation sequencing will help to develop a robust phylogenetic view of the tonsil community across geographically distant herds and commercially relevant species of pigs and management styles, and allow comparison of communities in healthy animals to those in animals with disease as well as asymptomatic carriers of pathogens.

## Conclusions

The 16S rRNA gene pyrosequencing results reported here extend and support our previous studies using 16S clone libraries to describe the microbial communities in tonsils of healthy pigs. Our results have defined a core microbiome found in tonsil specimens from two herds and at two time points from the same herd, and have also demonstrated the presence of minor components of the tonsillar microbiome unique to each herd. How the normal microbiota of the tonsils varies with and affects acquisition and carriage of pathogens, both porcine pathogens and those associated with foodborne illness in humans, is the subject of ongoing studies.

## Authors' contributions

BAL, TLM, and MHM contributed to the design of the study, performed the data analyses, and wrote the manuscript; NIC prepared and processed the DNA samples; RNK designed the tonsil brushes and performed all veterinary procedures; MK performed necropsies and collection of specimens. All authors read and approved the final manuscript.

## Supplementary Material

Additional file 1 **Complete list of all phyla identified**. This is an Excel file listing all phyla identified in each pig tonsil sample and the number of unique sequences belonging to each phylum within each sample, in descending order of frequency found in the total data set. Horizontal divisions indicate phyla found in all samples, those found in Herd 2 only, and those found in Herd 1 only. Phyla that comprise the core microbiome are highlighted. Click here for file

Additional file 2 **Complete list of all classes identified**. This is an Excel file listing all classes identified in each pig tonsil sample and the number of unique sequences belonging to each class within each sample, in descending order of frequency found in the total data set. Horizontal divisions indicate classes found in all samples, those found in Herd 2 only, and those found in Herd 1 only. Classes that comprise the core microbiome are highlighted. Click here for file

Additional file 3 **Complete list of all orders identified**. This is an Excel file listing all orders identified in each pig tonsil sample and the number of unique sequences belonging to each order within each sample, in descending order of frequency found in the total data set. Horizontal divisions indicate orders found in all samples, those found in Herd 2 only, and those found in Herd 1 only. Orders that comprise the core microbiome are highlighted. Click here for file

Additional file 4 **Complete list of all families identified**. This is an Excel file listing all families identified in each pig tonsil sample and the number of unique sequences belonging to each family within each sample, in descending order of frequency found in the total data set. Horizontal divisions indicate families found in all samples, those found in Herd 2 only, and those found in Herd 1 only. Families that comprise the core microbiome are highlighted. Click here for file

Additional file 5 **Complete list of all genera identified**. This is an Excel file listing all genera identified in each pig tonsil sample and the number of unique sequences belonging to each genus within each sample, in descending order of frequency found in the total data set. Horizontal divisions indicate genera found in all samples, those found in Herd 2 only, and those found in Herd 1 only. Genera that comprise the core microbiome are highlighted. Click here for file
